# Recent progress in alkynylation with hypervalent iodine reagents

**DOI:** 10.1039/d2cc06168f

**Published:** 2023-01-19

**Authors:** Eliott Le Du, Jérôme Waser

**Affiliations:** a Laboratory of Catalysis and Organic Synthesis, Institute of Chemical Sciences and Engineering École Polytechnique Fédérale de Lausanne EPFL, SB ISIC, LCSO, BCH 4306 1015 Lausanne Switzerland jerome.waser@epfl.ch

## Abstract

Although alkynes are one of the smallest functional groups, they are among the most versatile building blocks for organic chemistry, with applications ranging from biochemistry to material sciences. Alkynylation reactions have traditionally relied on the use of acetylenes as nucleophiles. The discovery and development of ethynyl hypervalent iodine reagents have allowed to greatly expand the transfer of alkynes as electrophilic synthons. In this feature article the progress in the field since 2018 will be presented. After a short introduction on alkynylation reactions and hypervalent iodine reagents, the developments in the synthesis of alkynyl hypervalent iodine reagents will be discussed. Their recent use in base-mediated and transition-metal catalyzed alkynylations will be described. Progress in radical-based alkynylations and atom-economical transformations will then be presented.

## Abstract 2

Although alkynes are one of the smallest functional groups, they are among the most versatile building blocks for organic chemistry. In this feature article, the progress in alkynylation with hypervalent iodine reagents since 2018 will be presented.

## Graphical abstract

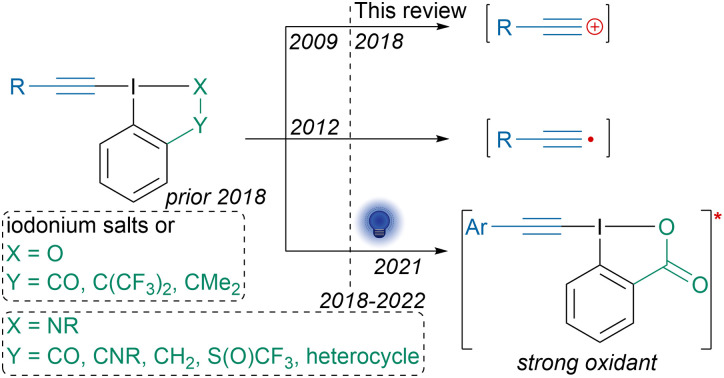

## 1. Introduction and context

Alkynes are highly versatile functional groups in organic chemistry, which also have found applications in applied fields such as biochemistry and material sciences.^1,2^ Among the main transformations that alkynes can undergo, the 1,3-dipolar cycloaddition with organic azides, also known as Huisgen cycloaddition or “Click Chemistry”, is of the utmost importance and was recognized with the Nobel prize in 2022.^3–5^ The high interest of the scientific community in alkynes has led to constant efforts to develop new flexible and efficient strategies to access them.

**Scheme 1:**
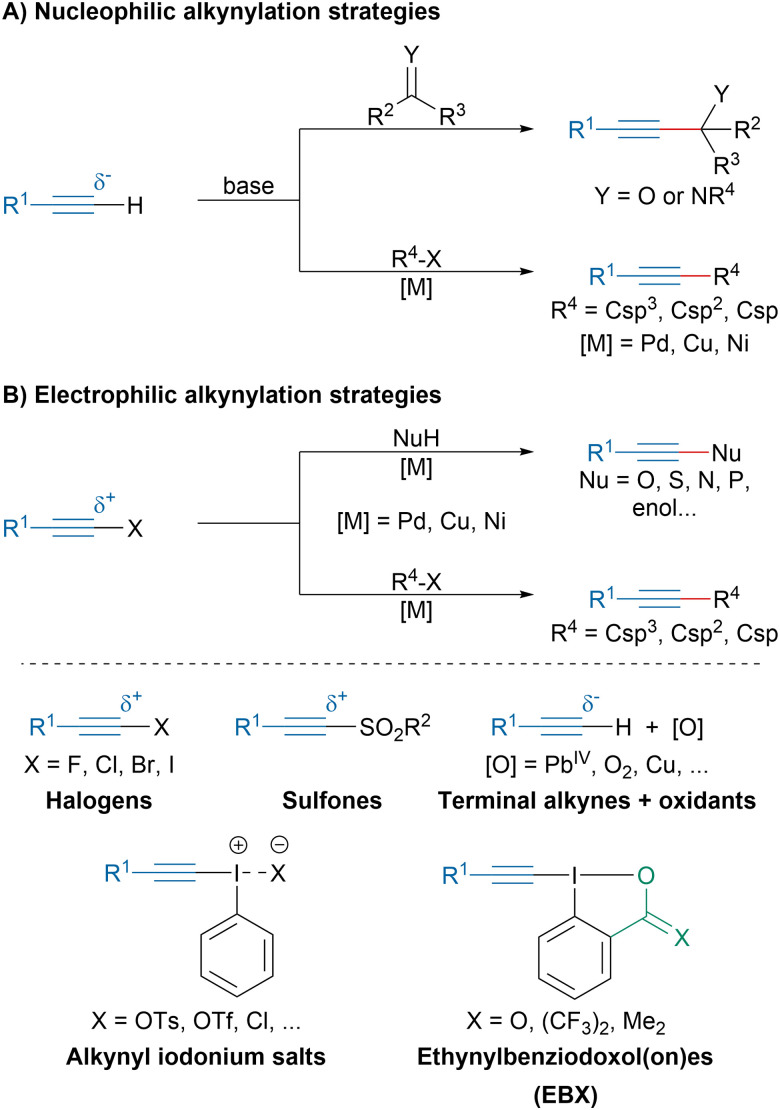
Nucleophilic and electrophilic alkynylation strategies.

Traditionally, most methods to access alkynes by transfer of a triple bond relied on the deprotonation of terminal alkynes generating nucleophilic acetylide intermediates that could then react with electrophiles (Scheme 1A). For instance, it is a method of choice to access propargylic alcohols or amines.^6,7^ Alternatively, acetylides can be involved in cross-coupling reactions such as the Sonogashira coupling,^8^ the Glaser dimerization or the Cadiot–Chodkiewicz reaction.^9^ While terminal alkynes are intrinsically nucleophilic, their reactivity can be reversed by installing an electron-withdrawing leaving group at an extremity (Scheme 1B).^10^ Initially, haloalkynes have been investigated in transition-metal catalyzed carbon–carbon and heteroatom–carbon couplings.^11^ Later, alkynyl sulfones have emerged as valuable partners for the alkynylation of nucleophilic radicals.^12^ The *in situ* oxidation of terminal alkynes has been also investigated but initially relied on toxic and highly reactive oxidants, which limited the application of these strategies.^10^ Since the discovery of the exceptional reactivity of the hypervalent bond, hypervalent iodine reagents have attracted the interest of synthetic chemists.^13–21^ In particular, alkynyl iodonium salts and ethynylbenziodoxolone (EBX) reagents have been particularly prolific as electrophilic alkyne synthons.^22–25^

**Scheme 2:**
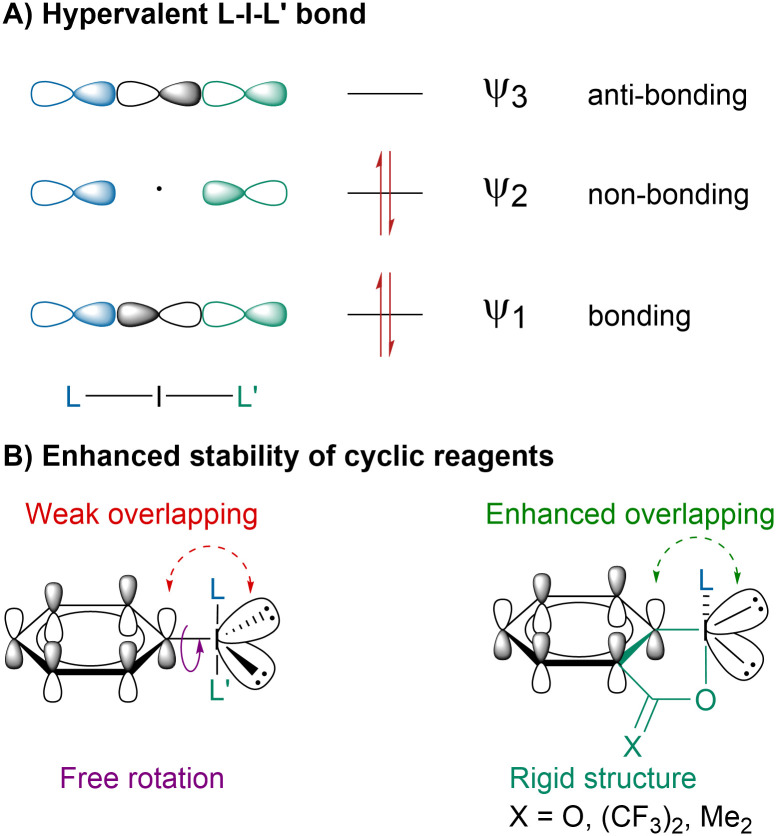
Hypervalent iodine bond and enhanced stability of cyclic reagents.

The peculiar reactivity of hypervalent iodine reagents arises from the 3-center-4-electron bond or hypervalent bond, which is longer, more polarized and weaker than a standard covalent bond leading to a higher electrophilic reactivity (Scheme 2A).^26,27^ Although the concept of hypervalency is still debated,^28–30^ it has been largely accepted to describe the unusual properties of hypercoordinated main-group elements. In recent years, most efforts have focused on cyclic hypervalent iodine reagents due to their higher stability (Scheme 2B).^31^ The additional stabilization has been proposed to arise from locking the iodine atom in an iodoheterocycle leading to an enhanced orbital overlapping.^32^ Moreover, the confinement of the oxygen lone pairs out of the 3c–4e plane disfavors the reductive elimination between the axial ligands.^33^

Although iodonium salts were initially investigated for the alkynylation of nucleophiles, their instability limited their wider application.^34,35^ Since 2009, bench stable EBX reagents have emerged as powerful electrophilic alkyne synthons in metal-free and metal-catalyzed alkynylation of transient radicals, heteroatom and carbon-centered nucleophiles.^31,36^ The purpose of this feature article is to present the progress in the field since our last reviews in 2018^24,25^ up to October 2022. The development of alkynyl hypervalent iodine reagents will first be described (Chapter 2). Then base-mediated (Chapter 3) and transition-metal mediated (Chapter 4) alkynylation reactions will be presented. Finally radical-based transfer of acetylenes (Chapter 5) and atom-economical transformations (Chapter 6) will be discussed.

## 2. Development of alkynyl hypervalent iodine reagents

**Scheme 3:**
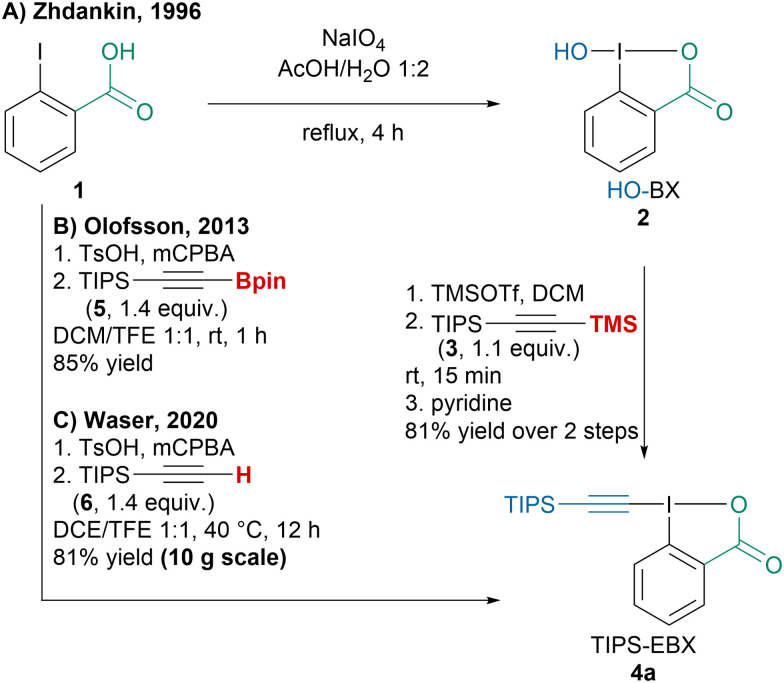
Improvements in the synthesis of EBX reagents (illustrated with TIPS-EBX (4a)).

Since the discovery of EBX reagents by the Ochiai group, efforts have focused on improving their synthesis.^37^ Zhdankin and coworkers reported a first general two-step procedure to access alkyl-, aryl- or silyl-substituted EBX reagents *via* hydroxybenziodoxole (2) (Scheme 3A).^38^ Following a renewed interest for these reagents, the Olofsson group developed a one-pot two-step procedure converting 2-iodobenzoic acid (1) into EBX reagents using pinacol alkynylboronates such as 5 (Scheme 3B).^39^ Interestingly this strategy could also be employed to access alkynyl iodonium salts. Our group later reported a similar protocol but using commercially available terminal alkyne 6 for the synthesis of TIPS-EBX (4a) on multigram scale (Scheme 3C).^40^ However, lower yields were obtained with alkyl or aryl substituted alkynes with this protocol and the Olofsson or Zhdankin protocols are usually preferred to synthesize them.

**Scheme 4:**
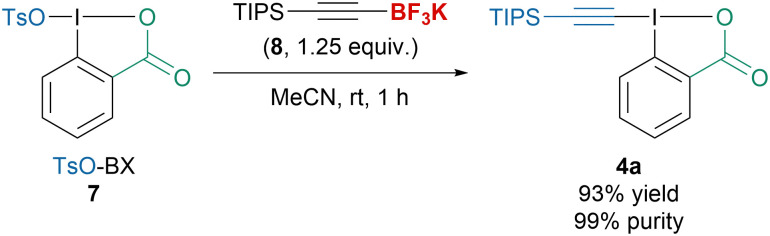
Synthesis of TIPS-EBX (4a) from TsO-Bx (7).

In 2022, our group disclosed a new procedure to access a broad variety of alkyl-, aryl- or silyl-substituted EBX reagents starting from TsO-BX (7) and more stable alkynyltrifluoroborates 8 (Scheme 4).^41^ The transformation tolerated a variety of solvents, did not require any additive and produced EBX reagents in high purity without purification, which allowed to directly apply them for the functionalization of different nucleophiles.

**Scheme 5:**
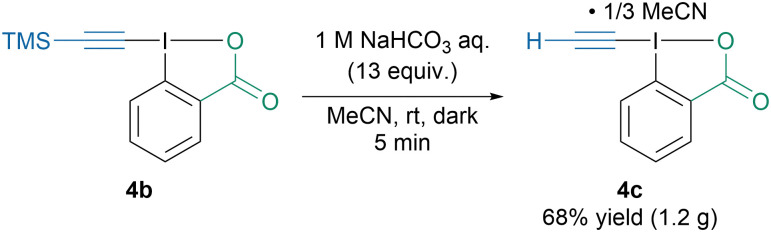
Synthesis of EBX-MeCN complex 4c.

Interestingly, Itoh, Tada and coworkers reported the synthesis of ethynylbenziodoxolone (4c) as a self-assembled double-layered honeycomb complex with MeCN, which allowed to isolate the otherwise highly unstable reagent (Scheme 5).^42^ Reagent 4c was then successfully used for the *N*-ethynylation of various sulfonamides and amino-acids. However, to avoid degradation of the reagent, all the reactions were run in the dark.

**Scheme 6:**
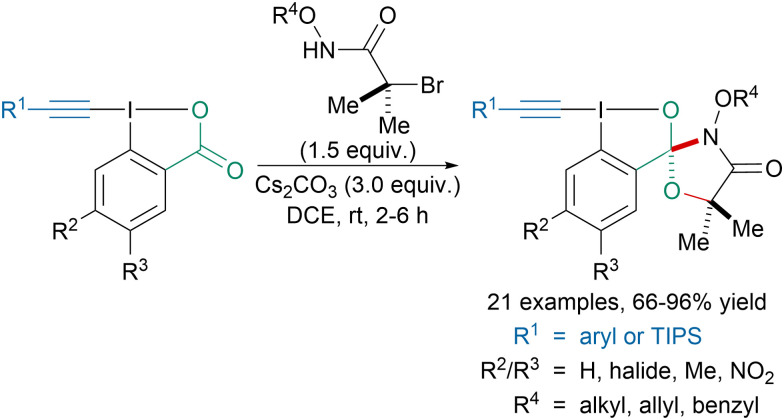
Synthesis of spirocyclic alkynyl hypervalent iodine reagents.

In 2021, Kang, Chen and coworkers reported the first synthesis of spirocyclic alkynyl hypervalent iodine reagents (Scheme 6).^43^ Instead of reacting with the alkyne to form ynamides or vinylbenziodoxolone reagents,^44^ under basic conditions, *N*-(oxy)-2-bromo-2-methylpropanamides reacted with the carbonyl group leading to spirocyclic reagents in good to excellent yields. These reagents could then be applied to the synthesis of benzoxazepine derivatives and diynes. Later, the spirocyclic reagents were used to access aryl ethers and thioethers.^45,46^

**Scheme 7:**
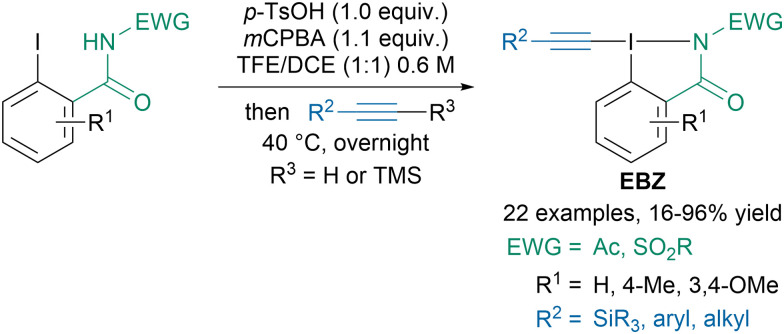
Synthesis of ethynylbenziodazolone (EBZ) reagents.

With the aim of synthesizing α-alkynyl amino acid derivatives, our group developed a new class of reagents: ethynylbenziodazolone (EBZ) bearing an amide instead of a carboxylic acid in the iodoheterocycle (Scheme 7).^47^ The reagents were readily accessed *via* a one-pot two-step procedure from the corresponding iodobenzamide and either terminal or TMS-substituted alkynes. The developed regents exhibited similar reactivity as standard EBX reagents in alkynylation of β-ketoesters, thiols or indoles.

**Scheme 8:**
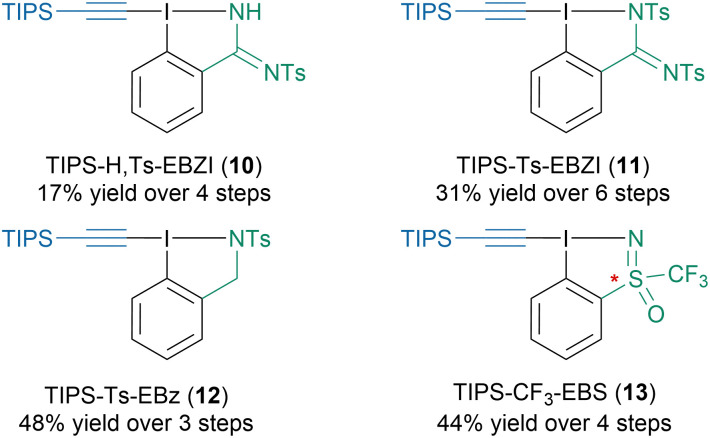
N-Heterocyclic alkynyl hypervalent iodine reagents developed in the Waser group.

As EBX reagents offer only limited possibilities for reactivity fine-tuning *via* structure modification, our group developed new N-heterocyclic reagents with increased structural flexibility (Scheme 8).^48^ For instance, we could synthesize mono- (10) and bis-protected (11) amidine based reagents as well as ethynylbenziodazole reagent (12) from 2-iodobenzonitrile. Through a collaboration with the Magnier group we could also access a racemic and an enantiopure sulfoximine based reagent (13). Unfortunately, they exhibited lower reactivity than the benchmark TIPS-EBX reagent (4a) in the reactions tested to date. More recently, the Nachtsheim group developed N-heteroaromatic alkynyl reagents.^49^

## 3. Base mediated alkynylation reactions

### 3.1. Alkynylation of C-nucleophiles

**Scheme 9:**
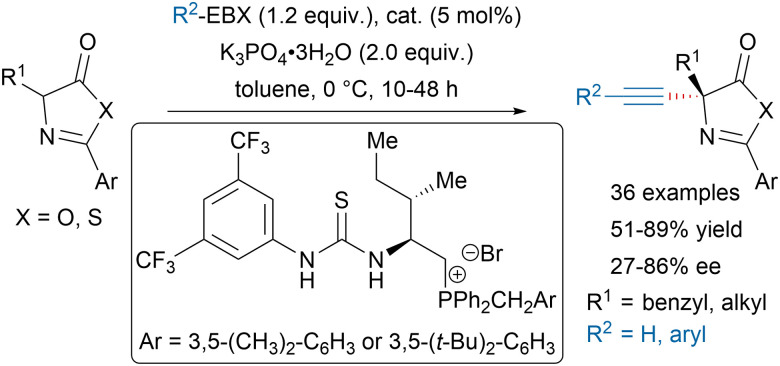
Asymmetric alkynylation of azlactones and thiazolones.

Since their first applications in electrophilic alkynylation of enolates,^50,51^ hypervalent iodine reagents have been widely used to install alkynes on the α-position of carbonyls, especially in total synthesis.^52–58^ In addition, the Bisai group recently reported a transition-metal free alkynylation of 2-oxindoles using EBX reagents.^59^ Using thiourea phosphonium salt catalysts, Wu and coworkers could develop an asymmetric alkynylation of azlactones and thiazolones leading to precursors of quaternary α-amino acids (Scheme 9).^60^

In addition, the Teodoro and Silva reported a protocol to access α-alkynyl β-substituted ketones by trapping an enolate, generated *via* Michael addition, with EBX reagents.^61^

**Scheme 10:**
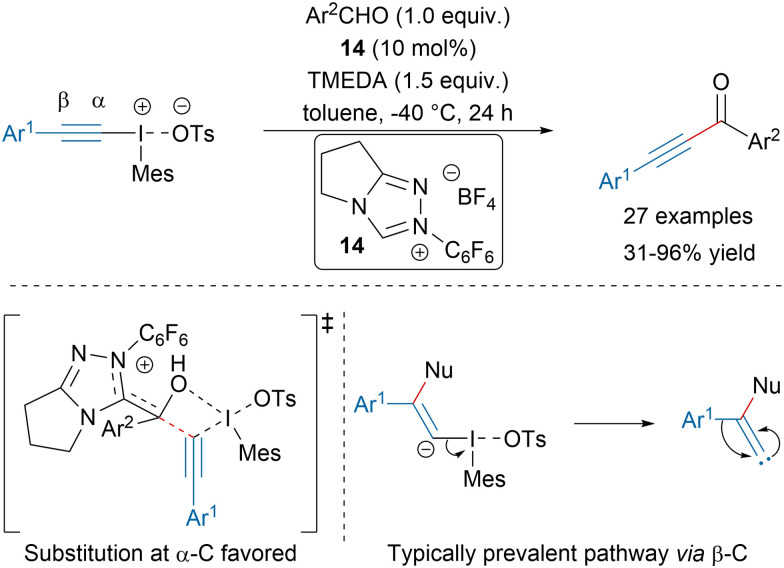
NHC-catalyzed synthesis of ynones.

In 2020, the Kalek group disclosed a N-heterocyclic carbene (NHC) catalyzed alkynylation of aromatic aldehydes with iodonium salts leading to ynones in moderate to excellent yields (Scheme 10).^62^^13^C-labelling experiments and computational mechanistic studies revealed that the reaction might proceed *via* direct substitution at the α-acetylenic carbon. This report represented the first example in which this mechanism was significantly favored for a C-centered nucleophile compared to the typically prevalent pathway involving initial attack at the β-position. Such addition would lead to a vinylidene carbene, which upon 1,2-shift would provide the product as originally proposed by the Ochiai group.^63^

M. Waser and coworkers developed a transition-metal free Cadiot–Chodkiewicz coupling of terminal alkynes and EBX reagents leading to unsymmetrical 1,3-diynes.^64^ Gold-catalysis had previously been shown to be able to promote this transformation as well.^65–67^

### 3.2. Alkynylation of N-nucleophiles

**Scheme 11:**
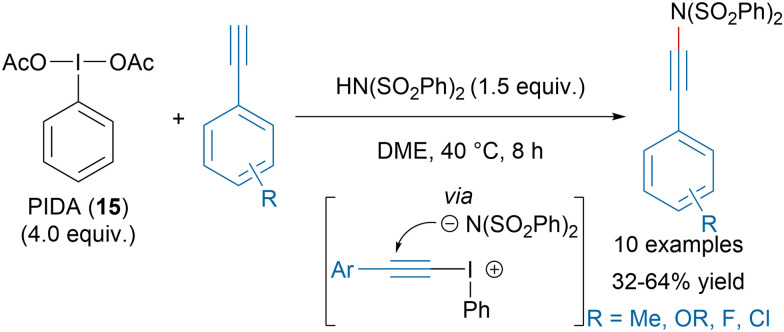
Ynamide synthesis with *in situ* generated alkynyl iodonium salts.

The formation of N–Csp bonds has attracted a lot of attention in the last decades due to the high versatility of ynamides.^68–70^ Hypervalent iodine reagents have emerged as valuable tools especially for nitrogen nucleophiles reacting poorly in copper-catalyzed alkynylation transformations. Li, Zhang and coworkers recently reported a metal-free synthesis of ynamides with *in situ* generated alkynyl iodonium salts from PIDA (15) and dibenzylsulfonimide (Scheme 11).^71^

**Scheme 12:**
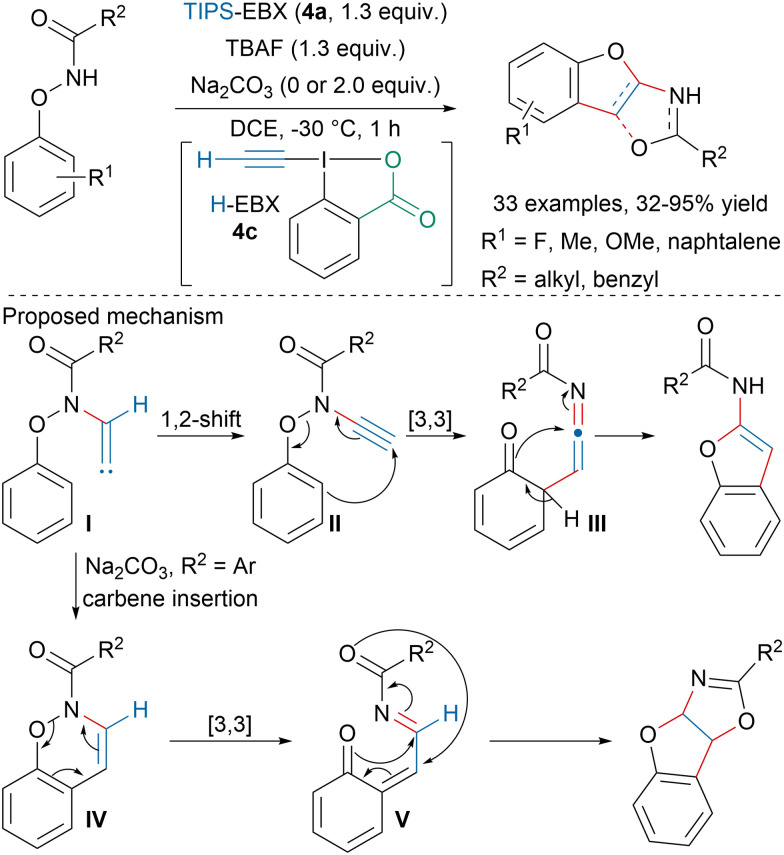
Synthesis of benzofuran derivatives from EBX and *N*-phenoxyamides.

Building upon their previous works on the formation of heterocycles with EBX reagents,^72,73^ Wen and coworkers developed a TBAF-mediated synthesis of benzofuran derivatives from *N*-phenoxyamides and TIPS-EBX (4a) (Scheme 12).^74^ When R^2^ is an alkyl, *in situ* formed H-EBX (4c) would be trapped by the *N*-phenoxyamide to form ynamide II after 1,2-hydride shift from intermediate I. [3,3]-Rearrangement followed by cyclization would lead to benzofurans. In contrast, when R^2^ is an aryl and in presence of a Na_2_CO_3_, vinylidene carbene I would undergo carbene insertion on the aryl ring followed by [3,3]-rearrangement, Michael addition and cyclization.

### 3.3. Alkynylation of S-nucleophiles and application to the functionalization of biomolecules

**Scheme 13:**
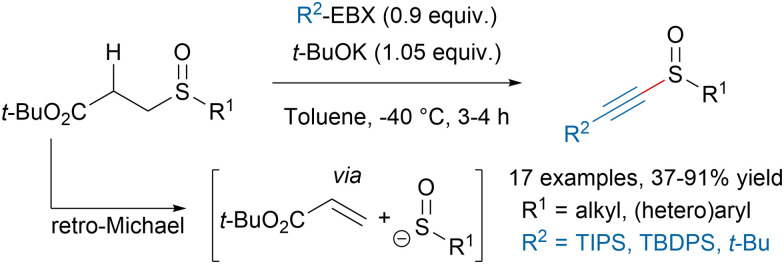
Metal-free synthesis of alkynyl sulfoxides.

Alkynyl sulfoxides are valuable building blocks in organic chemistry with unique reactivities. However the lack of robust procedures to synthesize them has limited their wider applications. In 2019, our group disclosed a new method to access alkynyl sulfoxides from EBX reagents and sulfenate anions formed *in situ via* a retro-Michael reaction (Scheme 13).^75^ This protocol allowed to avoid the use of strong oxidants often leading to overoxidation, as well as nucleophilic alkyne derivatives, which could react with the products through 1,4-additions.

**Scheme 14:**
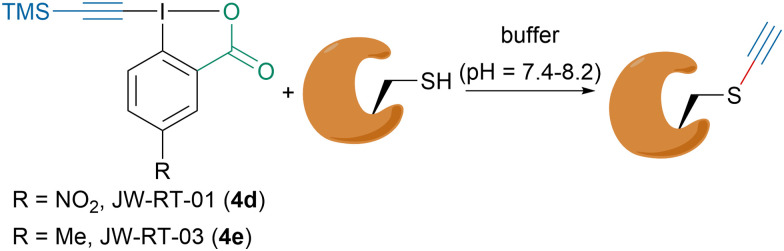
Ethynylation of cysteine residues with EBX 4d and 4e.

Owing to their high reactivity and biocompatibility, hypervalent iodine reagents have recently emerged as powerful tools for the functionalization of biomolecules.^76^ In the last decade, our group has extensively studied the alkynylation of thiols and especially of cysteines with the goal to functionalize biomolecules.^77–79^ Interestingly, our group showed that depending on the reaction conditions vinylbenziodoxolone (VBX) formation could also occur, which led to the development of a “doubly orthogonal” labeling of peptides with EBX reagents.^80^ Moreover, in 2020, in collaboration with the Adibekian and Chaubet groups, we reported a method to ethynylate cysteine residues on peptides and proteins *in vitro* and in living cells (Scheme 14).^81^ It was found that under slightly basic buffer conditions TMS-EBX reagents JW-RT-01 (4d) and JW-RT-03 (4e) would be desilylated *in situ* generating the corresponding highly reactive H-EBX reagents that could be trapped by cysteine residues. JW-RT-03 could efficiently alkynylate cysteines in both HeLa lysates *in vitro* and in living cells, showing that this reagent could be used for cysteine proteomic profiling. TMS-EBX (4b) was also evaluated for the bioconjugation of the bioactive antibody trastuzumab and showed promising reactivity.

**Scheme 15:**
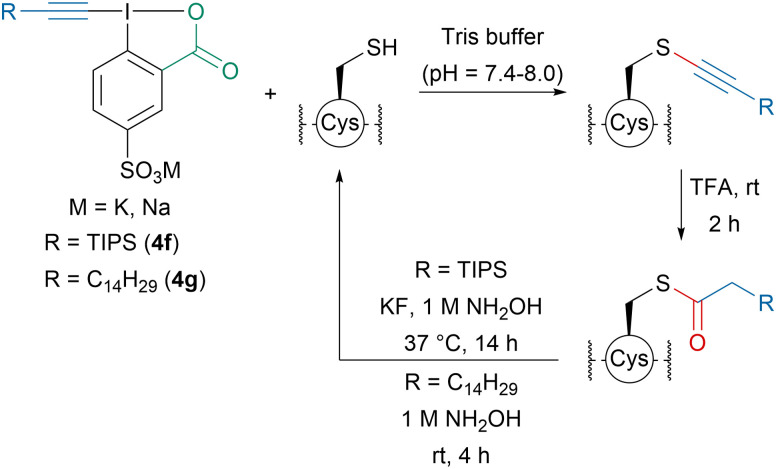
Lipophilization of peptides in water using amphiphilic reagents 4f and 4g.

Later, our group developed amphiphilic EBX-reagents 4f and 4g for the lipidation of cysteine residues (Scheme 15).^82^ The introduction of a sulfonate group on the aromatic ring favored water solubility. Peptides up to 18 amino acids as well as His_6_-Cys-Ubiquitin could be alkynylated in buffer and the modified peptides showed increased lipophilicity. Using TFA, the thioalkynes could be converted into thioesters, which could be cleaved in the presence of hydroxylamine regenerating the initial peptides.

**Scheme 16:**
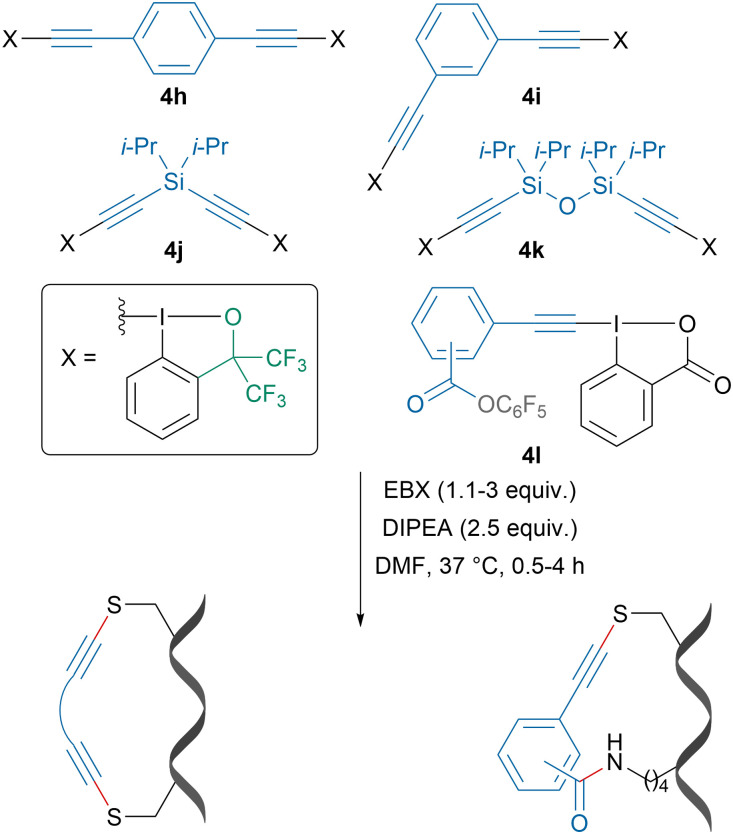
Cys–Cys and Cys–Lys stapling using bifunctional EBX reagents.

Leveraging the exquisite selectivity for cysteine alkynylation with EBX reagents, our group developed bifunctional reagents 4h–l, which could be used for i,i + 4 and i,i + 7 cysteine–cysteine and cysteine–lysine stapling of peptides (Scheme 16).^83^ Depending on the linker, changes in helicity were observed. A stapled peptide derived from the p53 protein showed increased helicity and binding affinity to MDM2 protein, a known cancer target and native binder to the p53 protein.

**Scheme 17:**
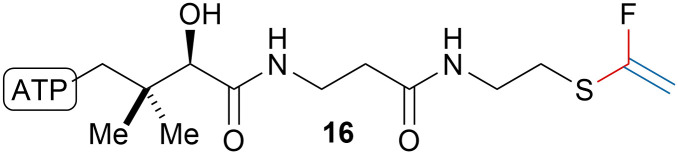
Fluorovinyl thioether acetyl coenzyme A analogue 16.

Building upon their work on the fluorination of thioalkynes,^84^ O’Hagan, Bühl and coworkers synthesized fluorovinyl thioether acetyl coenzyme A analogue 16 (Scheme 17).^85^ The fluorovinyl thioether moiety was obtained by alkynylation of a thiol using TMS-EBX (4b) followed by treatment of the obtained thioalkyne with AgF/I_2_/triethylamine. The compound was shown to be a potent inhibitor of citrate synthase (*K*_i_ = 4.3 μM).

In collaboration with the Matile group, several cyclic hypervalent iodine reagents were investigated as irreversible covalent inhibitors of thiol-mediated uptake. Although some showed promising activity, they usually exhibited early onset of toxicity.^86^

## 4. Transition-metal mediated alkynylation reactions

### 4.1. Alkynylation of metal-complexes

**Scheme 18:**
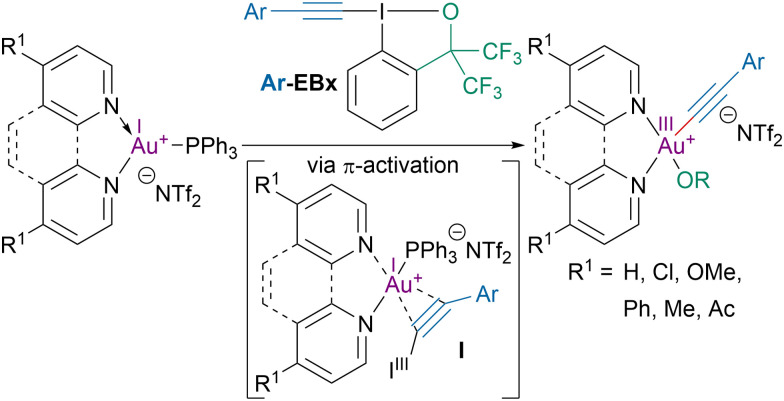
Oxidative addition of EBx reagents to gold(i) complexes.

In 2019, the Hashmi group investigated the role of the *trans*-influence of ligands on the oxidative addition of ethynylbenziodoxole (EBx) reagents to gold(i) complexes (Scheme 18).^87^ The oxidative addition was initiated by the formation of a π-interaction complex I between the alkyne and the gold catalyst. Mechanistic studies showed that the lower the σ-donating ability of the ligands was, the higher the rate of oxidative addition was. The increase in oxidative addition rate was attributed to an enhanced accessibility of gold(i) intermediate for the oxidizing reagent with less σ-donating ligands.

### 4.2. N–Csp bond formation

**Scheme 19:**
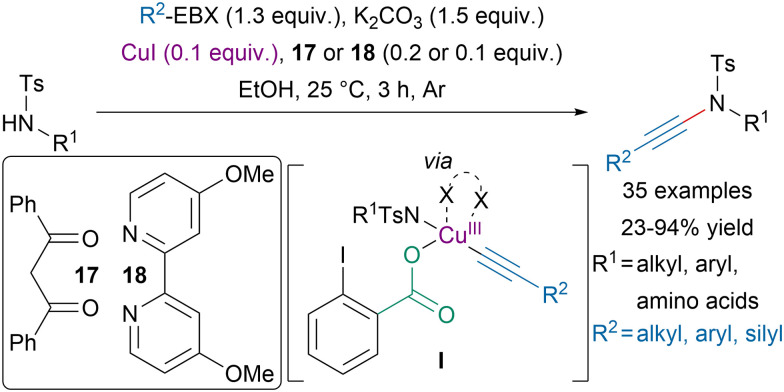
Copper-catalyzed ynamide synthesis with EBX reagents.

In 2021, Itoh, Tada and coworkers reported a copper-catalyzed *N*-alkynylation of sulfonamides with EBX reagents at room temperature (Scheme 19).^88^ Interestingly, the transformation was amenable to the alkynylation of the *N*-terminus of amino-acids and dipeptides. Moreover, mechanistic studies suggested that an electron-rich ligand, such as 17 or 18, and a protic solvent were required to reach high efficiency and promote the formation of oxidative addition intermediate I, allowing alkynes with bulky substituents to be used and preventing homodimerization.

**Scheme 20:**
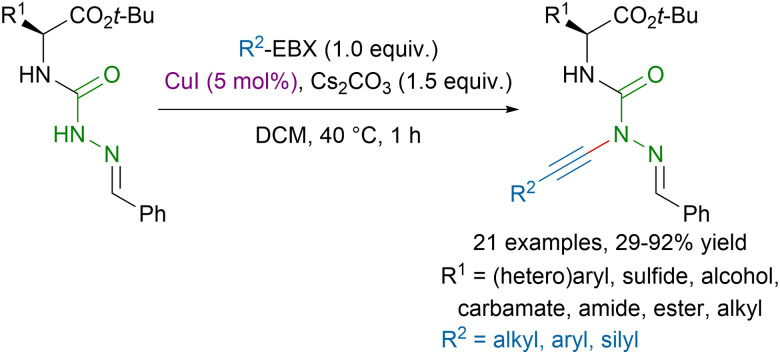
Copper-catalyzed alkynylation of azadipeptide derivatives.

While amides have been largely studied in alkynylation reactions, examples with hydrazides are scarce. The most popular method relied on the addition of acetylides on symmetrical diazodicarboxylates under strongly basic conditions.^89^ In 2022, our group reported a milder procedure for the direct alkynylation of hydrazides using copper-catalysis (Scheme 20).^90^ This method allowed to access functionalized azapeptide derivatives in moderate to excellent yields, tolerating a broad range of functional groups.

### 4.3. Aryl–Csp bond formation

**Scheme 21:**
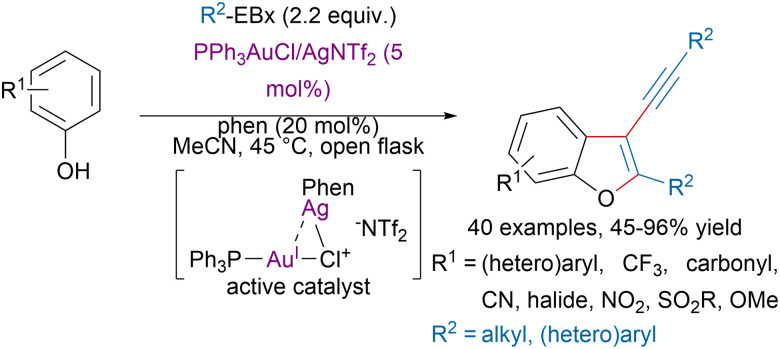
Synthesis of 3-alkynyl benzofurans with a bimetallic Au–Ag catalyst.

Over the last two decades, the combination of hypervalent iodine reagents with gold,^91^ and other transition-metals has allowed the functionalization of a broad range of substrates.^21^ For example, the Hashmi group reported a bimetallic gold–silver catalytic system for the synthesis of 3-alkynyl benzofurans from phenols and EBx reagents (Scheme 21).^92^ Mechanistic studies, suggested that a bimetallic Au–Ag catalyst promoted a tandem *ortho* C(sp^2^)–H alkynylation/oxyalkynylation reaction by leveraging the exceptional redox property and carbophilic π-acidity of gold. Based on a similar approach, Hashmi and coworkers also developed tandem C(sp^3^)–H alkynylation/oxyalkynylation reactions to access tetra-substituted furans from acceptor-substituted carbonyl compounds and indolizines from *ortho*-substituted pyridine derivatives.^93,94^

**Scheme 22:**
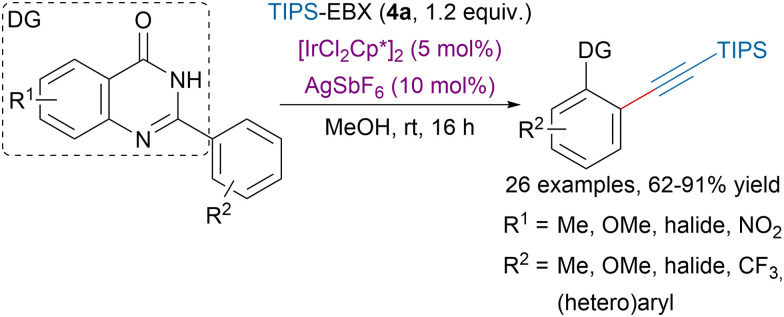
Arylquinazolin-4-ones directed alkynylation of (hetero)aryls.

In order to ensure high regioselectivity in C–H bond functionalizations the use of directing groups (DG) has become very popular, including in alkynylation transformations.^95^ Rohokale and coworkers developed an iridium-catalyzed *ortho* alkynylation of (hetero)aryls using TIPS-EBX (4a) and arylquinazolin-4-ones as directing groups (Scheme 22).^96^ Interestingly, switching the solvent for DCE and increasing the temperature to 70 °C allowed to access dialkynylated compounds in moderate to excellent yields.

**Scheme 23:**
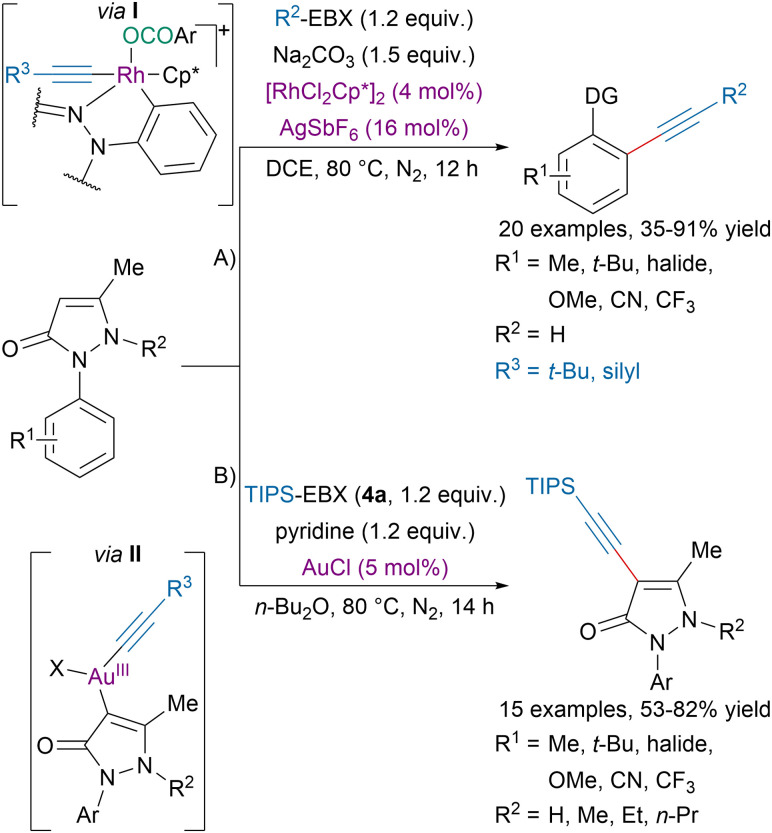
Regiodivergent alkynylation of 1-arylpyrazolones.

Alternatively, Xia, Zhang and coworkers developed a formal regiodivergent alkynylation of 1-arylpyrazolones (Scheme 23).^97^ With NH-free pyrazolone (A), a rhodium catalyst promoted a directed *ortho* C–H alkynylation *via* the proposed rhodacycle intermediate I. On the other hand, pyrazolones with alkylated nitrogen underwent C4-alkynylation under gold-catalysis *via* the proposed intermediate II (B). The authors suggested that the regioselectivity was determined by the nature of the substrate (alkylated pyrazolone or not) and the choice of metal catalyst.

**Scheme 24:**
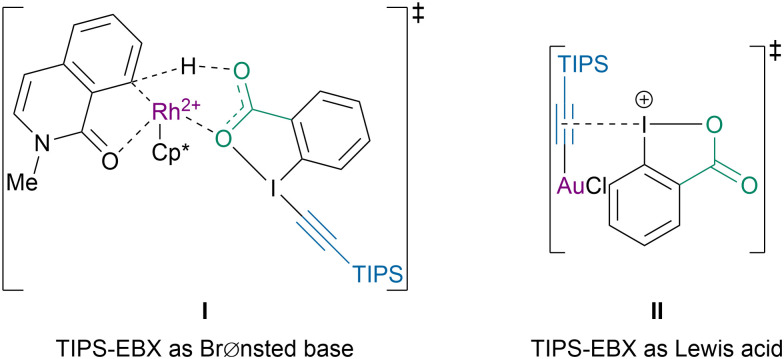
Dual reactivity of TIPS-EBX (4a) with Rh and Au catalysts.

A similar reactivity had been previously observed for the C–H alkynylation of *N*-methylisoquinolones.^98^ Computations showed that the difference of mechanism could also be explained by the dual reactivity of TIPS-EBX (4a) (Scheme 24).^99^ With the rhodium catalyst it behaved as a BrØnsted base, *via*I, favoring a base-assisted concerted metalation-deprotonation (CMD) mechanism for the C8–H bond activation. In contrast, under gold-catalysis the iodine(iii) center acted as a Lewis acid, *via*II, to activate the alkyne. The computations suggested that in this case steric hindrance, rather than electronic effects, directed the regiochemistry of the reaction.

Hypervalent iodine reagents have been also widely used for the functionalization of heterocycles.^100^ Since our initial report on the alkynylation of indoles and pyrroles with EBX reagents using gold-catalysis,^101^ several researchers investigated this transformation. For instance, the Liu group reported in 2018 a Ru(ii)-catalyzed C2-alkynylation of indoles using pyrimidine as a directing group.^102^ Using ball milling, Bolm and coworkers developed solventless alkynylations of indoles with either Rh(iii)-(C2-selective) or Au(i)-catalysis(C2 or C3 selective).^103^ The mechanochemical conditions allowed to reduce the reaction time and the catalyst loading without requiring additional heating, still maintaining excellent functional group tolerance. Dai, Bai, Ma and coworkers developed a directed C2-alkynylation of indoles and used the triple bond as a handle for further functionalization.^104^ Using AuCl for the C2 or C3-alkynylation of pyrroles, Furuta, Ishida and coworkers could access functionalized BODIPY dyes with distinctive spectroscopic properties.^105^

### 4.4. Alkene–Csp bond formation

**Scheme 25:**
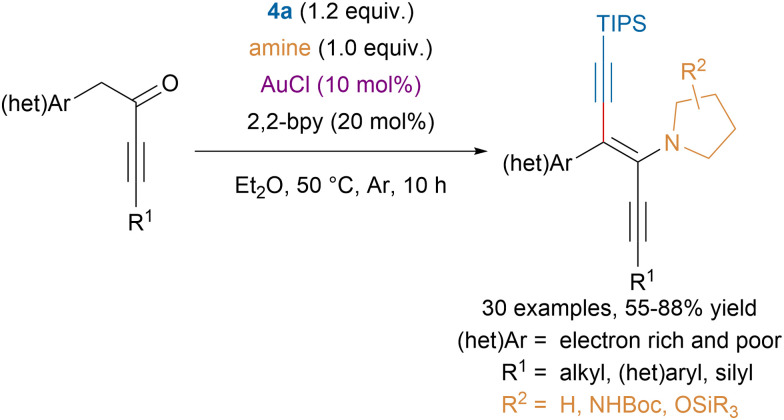
Gold-catalyzed diyneamines synthesis.

Building upon their previous works on synergistic gold-amine catalysis for the α-functionalization of carbonyls,^106^ the Huang group developed a procedure to access isolable diyneamines using gold catalysis (Scheme 25).^107^ The high conjugation of the π-system might explain why the corresponding ynones were not isolated. The alkynylation of enamines was also studied by the Hashmi group to access tetra-substituted 1,3-enynes from acceptor-substituted enamines.^108^

**Scheme 26:**
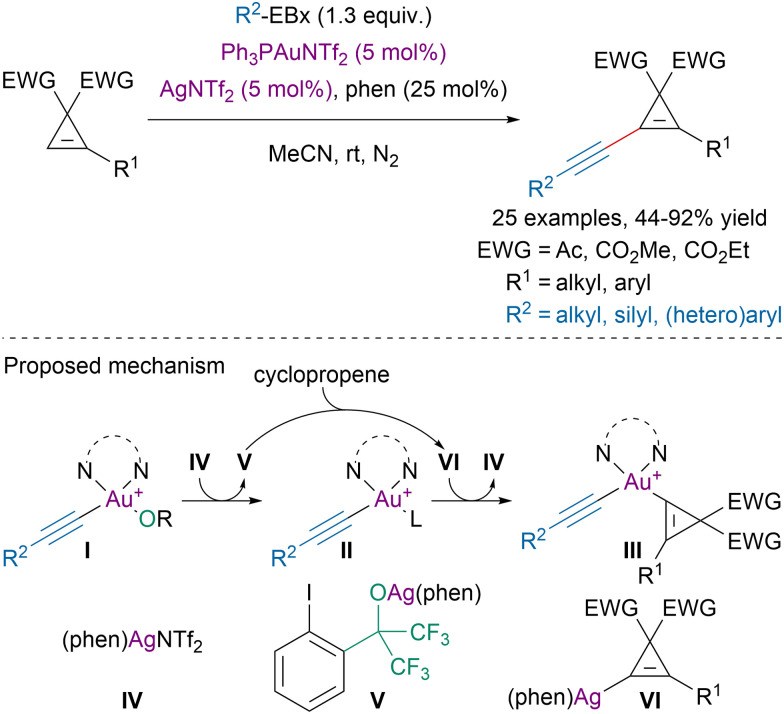
Dual Au/Ag-catalyzed alkynylation of cyclopropenes.

In 2019, Hashmi and coworkers disclosed a dual gold/silver-catalyzed direct alkynylation of cyclopropenes with ethynylbenziodoxole reagents (Scheme 26).^109^

Extensive mechanistic studies suggested that gold was involved in oxidative addition on EBx reagents leading to intermediate I. Ligand exchange with silver salt IV would lead to intermediates II and V. The later would be responsible for the C–H activation step affording intermediate VI. Transmetalation with intermediate II would generate III and reform the active silver catalyst IV. Reductive elimination from III would then provide the targeted alkynylated cyclopropene and close the gold-catalytic cycle.

**Scheme 27:**
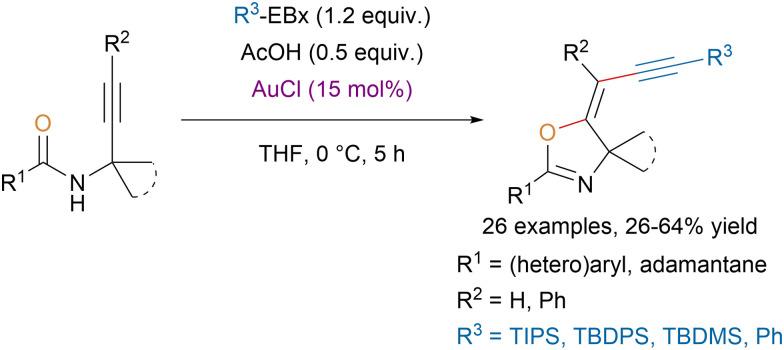
Au-catalyzed stereoselective synthesis of alkynyloxazolines.

The same group reported a stereoselective gold-catalyzed oxyalkynylation of *N*-propargylcarboxamides with EBx reagents leading to alkynyloxazolines (Scheme 27).^110^ The developed one-step procedure allowed to tolerate functional groups that would be prohibited for a Sonogashira coupling, which was traditionally used to access these scaffolds. Moreover, computations suggested that the observed stereoselectivity could be explained by kinetic control.

Alternatively, from propargyl alcohols, Patil, Senthilkumar and coworkers successfully developed a gold-catalyzed alkynylative Meyer-Schuster rearrangement affording enynones in moderate to excellent yields.^111^ The carbophilic π-acidity of gold was key for the success of the reaction as previous studies with palladium failed to promote an alkynylative Meyer-Schuster transformation.^112^

### 4.5. C(sp^3^)–Csp bond formation

**Scheme 28:**
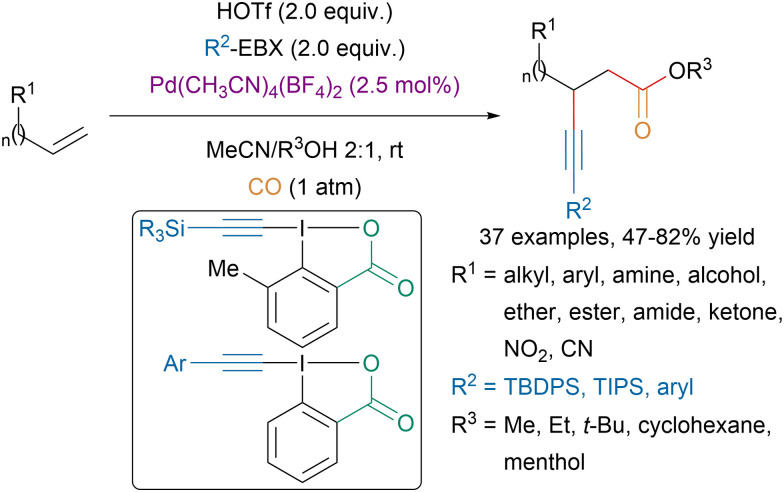
Pd-catalyzed alkynylcarbonylation of unactivated alkenes.

From simple unactivated alkenes, Liu and coworkers could access β-alkynylcarboxylic esters *via* a palladium catalyzed intermolecular alkynylcarbonylation (Scheme 28).^113^ The mild reaction conditions allowed a broad functional group tolerance and moderate to excellent regioselectivity were observed.

Moreover, mechanistic studies suggested that the reaction involved *cis*-addition of the alkynyl and the carbonyl moiety. Interestingly, for the transfer of silyl-substituted alkynes, reagents bearing a methyl group *ortho* to the iodine on the aromatic core led to better yield. This enhancement of reactivity, also called hypervalent twist, had previously been observed and first reported in oxidation reactions and in the gold catalyzed alkynylation of indoles.^114–116^

**Scheme 29:**
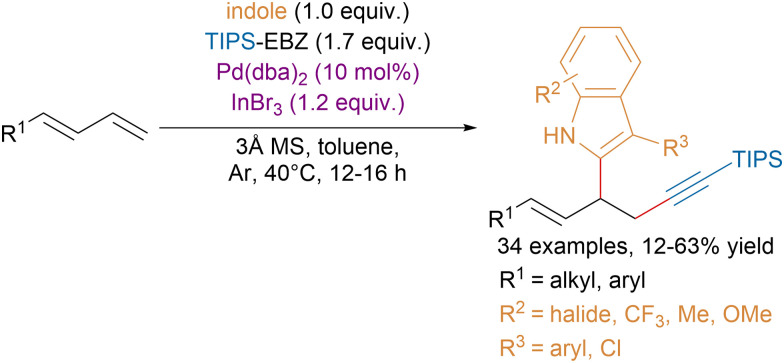
1,2-Functionalization of conjugated dienes with indoles and EBZ.

Recently, the Chen group reported a palladium-catalyzed three-component cross-coupling of 1,4-dienes with indoles and EBZ reagents (Scheme 29).^117^ Interestingly, they showed that this class of reagents outperformed EBX reagents, which produced mixture of 1,2- and 1,4-functionalization products.

**Scheme 30:**
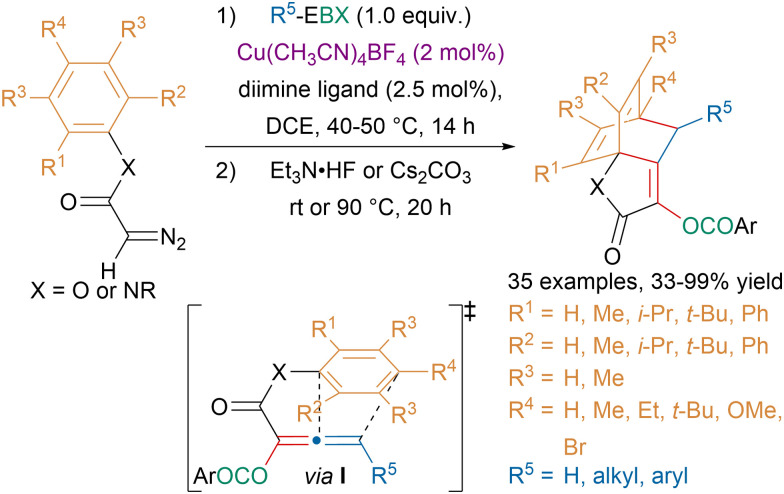
One-pot sequential oxyalkynylation/Himbert reaction.

In the last decade, the functionalization of diazo compounds with hypervalent iodine reagents has allowed to access numerous highly functionalized products.^118^ Our group took advantage of the ambiphilic reactivity of metal carbenes obtained from diazo compounds to develop atom-economical oxyalkynylation reactions.^119,120^ While studying product modifications, we discovered an unusual low-temperature [4 + 2]-cycloaddition of allenes and arenes, also known as Himbert reaction.^121^ Building upon this observation, our group reported a one-pot oxyalkynylation/Himbert reaction leading to bicyclo[2.2.2]octadiene products that could be used as diene ligands for rhodium catalysis (Scheme 30).^122^ The reaction is believed to involve first an oxyalkynylation of the diazo compounds. Then in the presence of fluoride or a base, an allene would be formed, which undergoes a Himbert cycloaddition. Computations suggested that the low activation energy for the cycloaddition arose from favorable dispersive interactions in the transition state I.

**Scheme 31:**
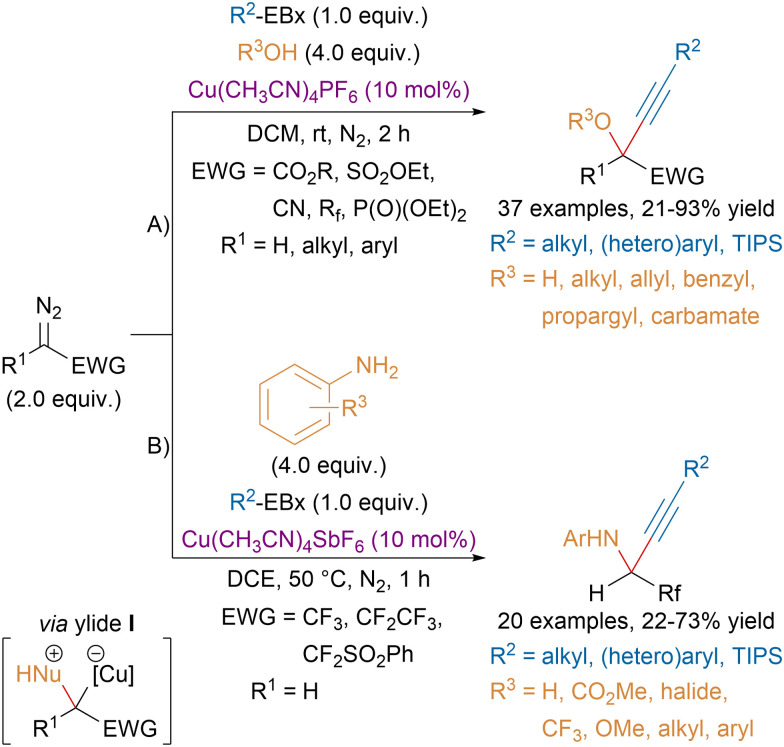
Cu-catalyzed *gem*-oxy- and aminoalkynylation of diazo compounds.

In order to increase structural diversity, our group later developed three-component reactions of diazo compounds, EBx reagents and alcohols or anilines (Scheme 31).^123,124^ The use of the benziodoxole core was critical for the success of the transformation as the corresponding fluorinated benzyl alcohol did not compete with external nucleophiles for the insertion in the metal carbene leading to ylide intermediate I. For the reaction with alcohols, a high structural diversity was achieved (A). On the other hand, the 3CR with amines has been limited so far to anilines and fluorinated diazo compounds (B).

## 5. Radical-based alkynylation reactions

Since the seminal work from Li, Cheng and coworkers,^125^ EBX reagents have emerged as powerful radical traps for alkynylation reactions. In the following chapter, progress in radical alkynylation transformations using hypervalent iodine reagents reported after our last review in the field will be presented.^36^

### 5.1. Non-photoredox-induced radical alkynylations

**Scheme 32:**
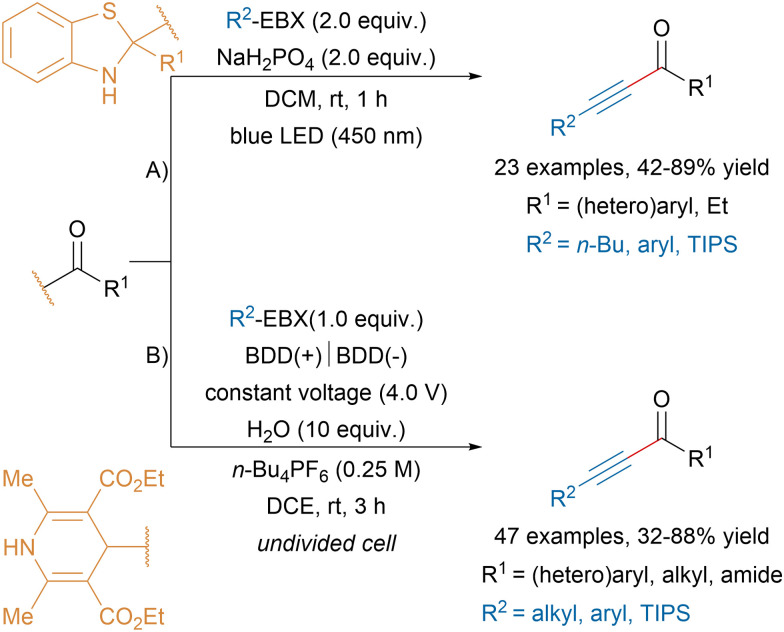
Ynone synthesis *via* radical alkynylation.

In 2019, Zhu and coworkers reported a visible light-induced alkynylation of acyl radicals leading to valuable alkyl and aryl ynones in moderate to good yields (Scheme 32A).^126^ The authors proposed that visible light irradiation of C2-acyl substituted benzothiazolines would promote a C–C bond homolytic cleavage leading to an acyl radical that would be trapped by EBX reagents. Similar products were obtained by the Maruoka group by heating aldehydes in presence of aryl ethynylbenziodoxole reagents, albeit in lower yields.^127^ Alternatively, the Wang group developed an electro-induced homolysis of 4-acyl-1,4-dihydropyridines generating acyl radicals that could react with EBX reagents (Scheme 32B).^128^ The mild reaction conditions allowed to access a broad range of ynones bearing functional groups and propiolamide derivatives. The late-stage functionalization of pharmaceutical molecules was also demonstrated.

**Scheme 33:**
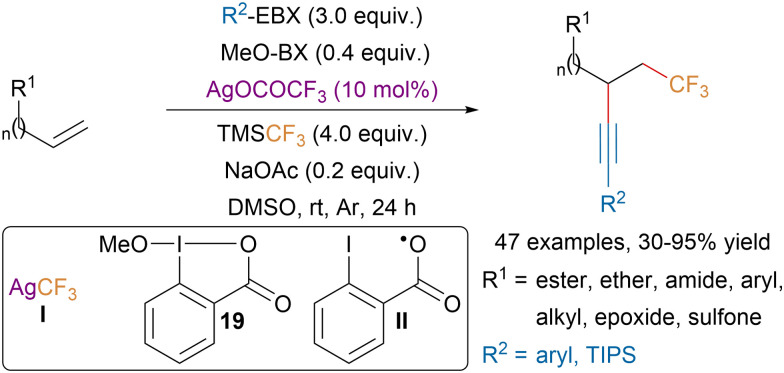
Ag-catalyzed trifluoromethylalkynylation of unactivated olefins.

In 2019, the Tsui group disclosed a silver-catalyzed trifluoromethylalkynylation of unactivated olefins tolerating a broad range of functional groups (Scheme 33).^129^ Mechanistic studies suggested that the reaction was proceeding through a radical mechanism with first addition of the trifluoromethyl radical and then trapping of the resulting nucleophilic radical by EBX reagents. NaOAc was required to initiate the formation of AgCF_3_ (I) which is responsible for the formation of CF_3_ radicals. MeO-BX (19) is believed to act as a source of iodanyl radical II, which is also generated after the radical alkyne transfer from EBX reagents. The authors proposed that radical II would oxidize AgCF_3_ to generate trifluoromethyl radicals.

**Scheme 34:**
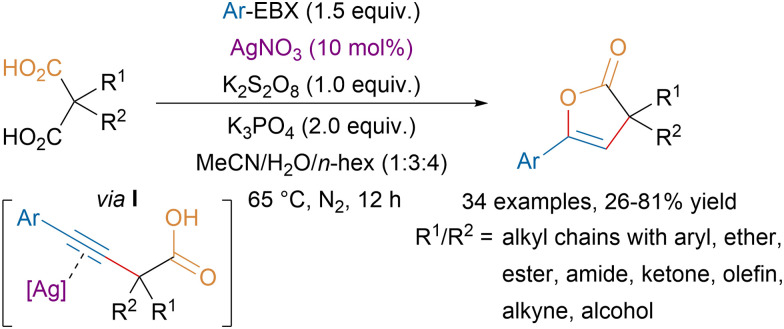
Ag-catalyzed monodecarboxylative alkynylation–lactonization of malonic acid derivatives.

Starting from malonic acid derivatives, the Chen group developed a tandem monodecarboxylative alkynylation–lactonization affording 2(3*H*)-furanones in moderate to good yields (Scheme 34).^130^ Key for the success of the transformation was the dual role of the silver catalyst, first promoting the decarboxylative alkynylation in presence of an oxidant and a base, then acting as a Lewis acid to activate the triple bond to catalyze the lactonization step *via* intermediate I.

Alternatively, using an ammonium persulfate oxidant, Xu, Huang and coworkers could access arylthiodifluoromethylated alkynes *via* the decarboxylative alkynylation of arylthiodifluoroacetic acids.^131^

### 5.2. Photoredox-catalyzed C–H functionalization

**Scheme 35:**
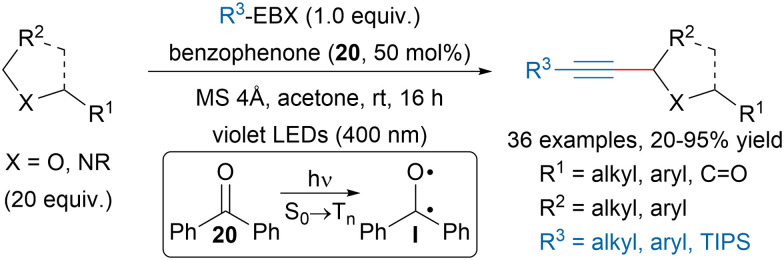
Benzophenone promoted C(sp^3^)–H alkynylation of ethers and amides.

In 2020, Nemoto, Nakajima and Matsumuto reported a benzophenone (20) promoted C(sp^3^)–H alkynylation of ethers and amides (Scheme 35).^132^ The use of violet LEDs (400 nm) allowed to selectively excite the photosensitizer and promote So → T_*n*_ transitions. The formed excited state I could then engage in CH abstraction on the substrate, which could then react with EBX reagents and the resulting iodanyl radical would then close the catalytic cycle.

In collaboration with our group, Kokotos and coworkers developed a similar transformation but using phenylglyoxylic acid and white CFL lamps.^133^ Interestingly, under the reaction conditions thioethers underwent deconstructive alkynylation affording thioalkynes.

**Scheme 36:**
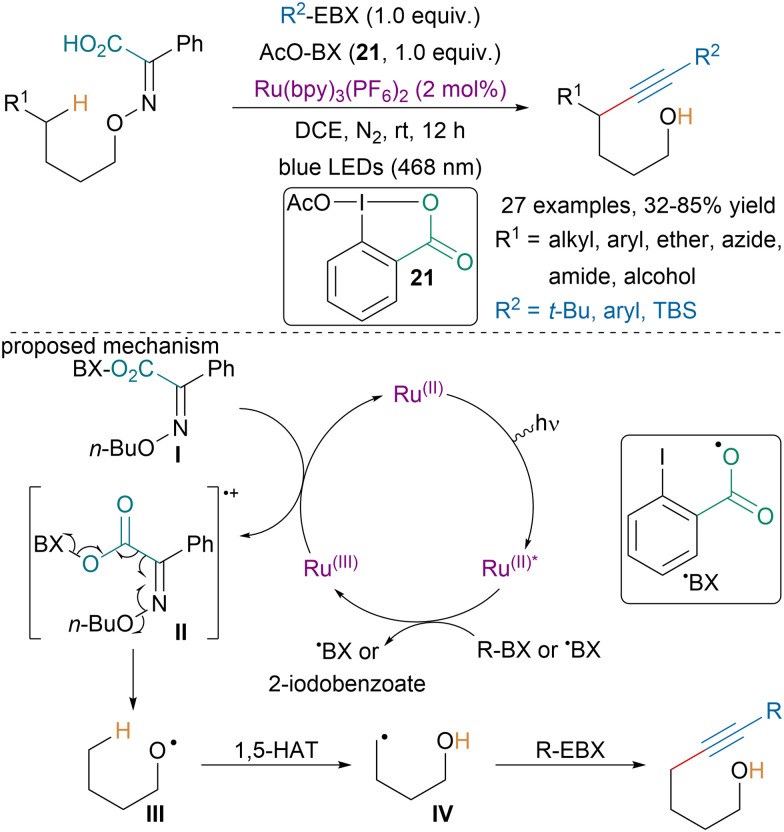
Photoredox-catalyzed remote C(sp^3^)–H alkynylation.

In 2022, the Chen group reported a photoredox-catalyzed remote C(sp^3^)–H alkynylation *via* the fragmentation of iminophenylacetic acids (Scheme 36).^134^ Based on literature precedence and mechanistic investigations, the authors suggested that first the substrate would engage in ligand exchange with 21 to afford intermediate I, which would be oxidized by a Ru(iii) species leading to II. Ru(iii) would be obtained after oxidative quenching of the excited-state Ru(ii)* by a hypervalent iodine intermediate. Fragmentation of II would lead to alkoxyl radical intermediate III, which upon 1,5-HAT would generate intermediate IV that could react with EBX reagents and generate the targeted compounds.

Furthermore, Hammond and coworkers showed that aryl-EBX reagents were efficient traps for alkyl radicals generated from 1,4-dihydropyridines under iridium photocatalysis.^135^

### 5.3. Photoredox-catalyzed π-system functionalization

**Scheme 37:**
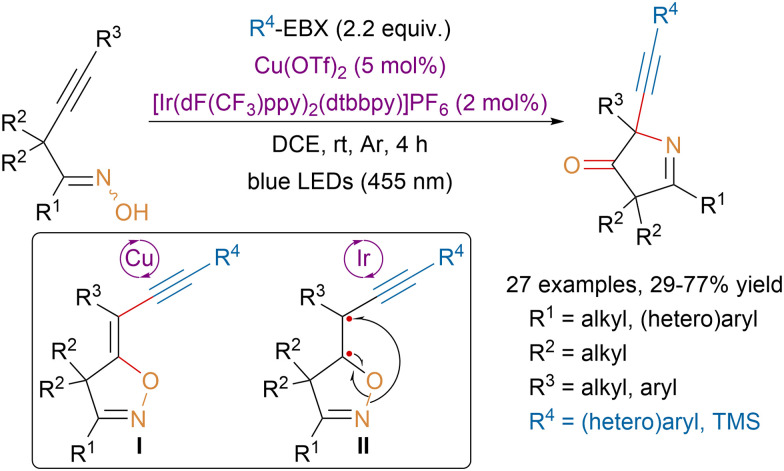
Synthesis of N-heterocycles *via* sequential Cu-catalyzed annulative alkynylation followed by Ir-catalyzed radical [1,4]-oxygen atom transfer.

In 2022, the Han group described sequential catalytic annulations for the synthesis of N-heterocycles *via* radical [1,4]-oxygen atom transfer from alkyne tethered ketoximes (Scheme 37).^136^ Mechanistic studies suggested that the first annulation would be catalyzed by copper and involve the generation of an oxygen-centered radical on the ketoxime. Radical addition on the alkyne followed by trapping of the resulting vinyl radical by EBX reagents would provide isolable intermediate I and an iodanyl radical that would close the catalytic cycle by oxidizing copper(i) back to copper(ii). Then triplet-state excitation of intermediate I by an iridium photocatalyst would generate diradical II, which would rearrange to afford the product *via* a [1,4]-oxygen atom transfer. Under Lewis acid catalysis a third annulation process was observed between the carbonyl group and the introduced alkyne.

### 5.4. Photoredox-catalyzed alkynylation *via* C–C bond cleavage

**Scheme 38:**
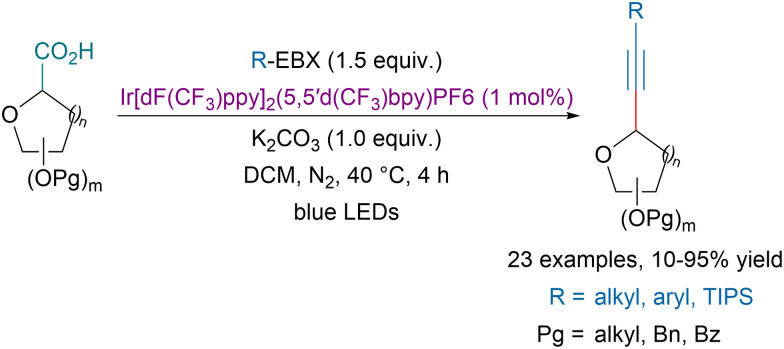
Photoredox-catalyzed decarboxylative alkynylation of glycosides.

In 2015, Xiao and our groups concurrently demonstrated that EBX reagents were valuable partners to develop decarboxylative alkynylation transformations.^137,138^ Zhang, Wang and coworkers showed that the transformation could also be catalyzed using recyclable graphitic carbon nitride polymers.^139^ Later our group could extend the concept to the photoredox catalyzed decarboxylative alkynylation of the C-terminus of peptides.^140^ Alternatively, in 2021, Zhang and coworkers reported a photoredox-catalyzed decarboxylative alkynylation of glycosides at the anomeric position (Scheme 38).^141^ A variety of alkynyl *C*-glycosides could be obtained in moderate to excellent yields with high diastereoselectivity.

**Scheme 39:**
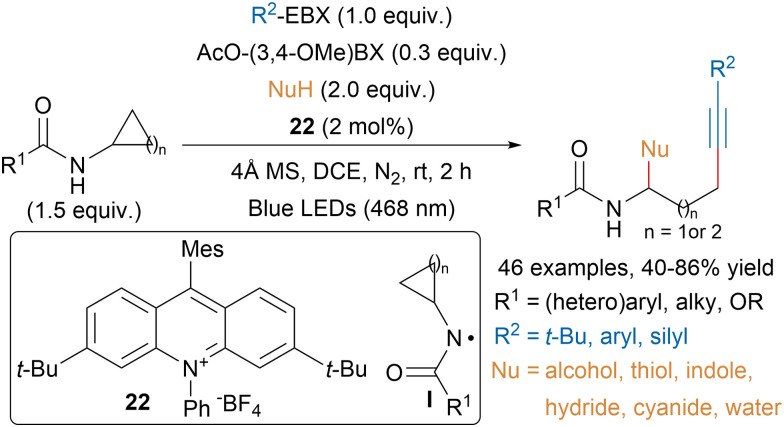
Photoredox-catalyzed alkynylative ring-opening of cycloalkylamides.

Chen and coworkers developed a photoredox-catalyzed ring opening alkynylation of cycloalkylamides using an acridinium photocatalyst (22) and hypervalent iodine reagents (Scheme 39).^142^ The authors proposed that the catalytic amount of AcO-(3,4-OMe)-BX reagent would facilitate the single-electron oxidation of cycloalkylamides leading to intermediate I. Then ring opening would generate an alkyl radical that could be trapped by EBX reagents and an imine that could react with nucleophiles.

### 5.5. Direct photo-excitation of Ar-EBX reagents

**Scheme 40:**
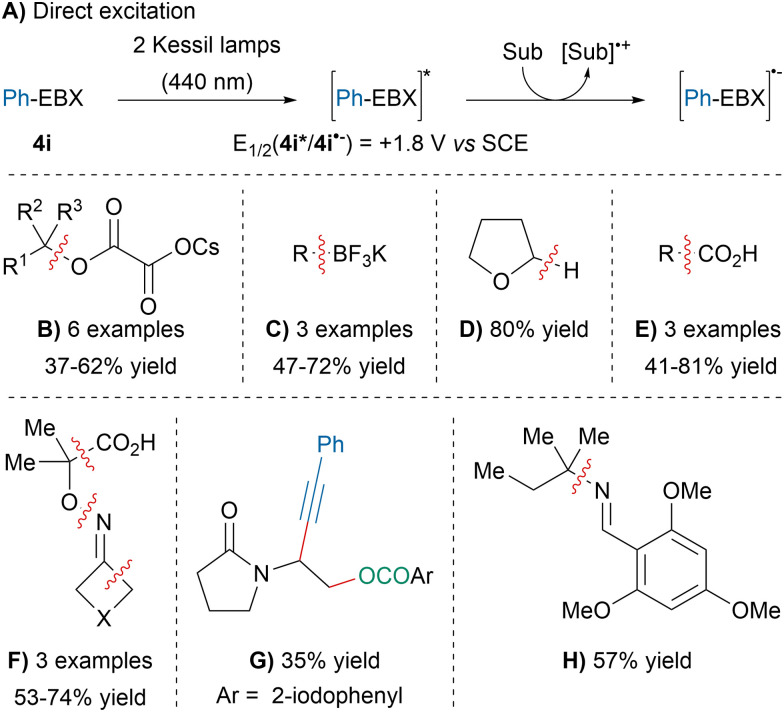
Direct photoexcitation of Ph-EBX with visible light and applications.

In 2021, our group discovered that the excited state of Ph-EBX (4i*), generated *via* direct light excitation using Kessil lamps, was a strong oxidant capable of oxidizing a broad range of substrate without the need for photocatalysts (Scheme 40A).^143^ For instance, the direct excitation of 4i promoted the deoxygenative alkynylation of cesium-oxalate salts (B). Slightly better yields were obtained with a photocatalyst after extensive optimization and was concurrently reported by the Xie group.^144^ The deboronative alkynylation of trifluoroborate salts developed by Chen and coworkers could also be initiated by direct excitation (C).^145,146^ In addition, 4i* was able to induce the alkynylation of THF (D) or the decarboxylative alkynylation of glyoxylic and aliphatic acids (E). The decarboxylative oxime fragmentation–alkynylation, previously described by our group,^147^ could be performed without the addition of organic dyes (F). Likewise, the atom-economical oxyalkynylation of enamides developed by our group could be promoted by the direct photoexcitation of Ph-EBX (4i) (G).^148^ Finally, an unprecedented deaminative-alkynylation of imines was also discovered using this approach (H).

**Scheme 41:**
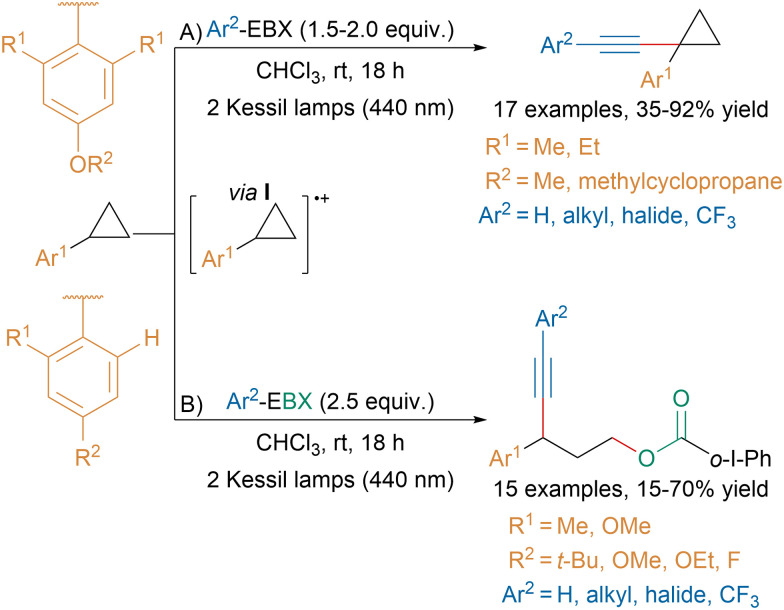
Photoexcitation of Ar-EBXs for the alkynylation of aryl cyclopropanes.

Building upon this work, our group recently developed a substrate-controlled C–H alkynylation or C–C oxyalkynylation of aryl cyclopropanes *via* the direct photoexcitation of Ar-EBX reagents (Scheme 41).^149^

When the aryl on the cyclopropane was bearing two *ortho* substituents, an unusual CH-alkynylation was taking place presumably *via* radical cation I (A). From this intermediate, computations suggested that the conformational constrains induced by the aryl ring favored H-abstraction followed by alkynylation over ring opening. In contrast, for aryl rings bearing only one *ortho* substituent, intermediate I underwent ring opening and then 1,3-oxyalkynylation (B). The same transformation had been previously reported by Studer and coworkers using an organic dye.^150^ Furthermore, our group showed that this reaction could be extended to the 1,3-oxyalkynylation of aminocyclopropanes and the 1,2-oxyalkynylation of styrenes.

## 6. Atom-economical transformations

**Scheme 42:**
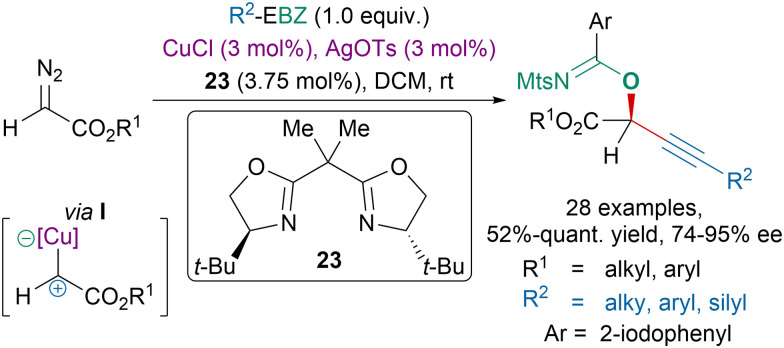
Copper-catalyzed oxyalkynylation of diazo compounds with EBZs.

The pursuit of greater efficiency in organic chemistry resulted in the development of atom economical reactions for which not only the alkyne of EBX reagents would be transferred but also the carboxylate moiety.^151,152^ For instance, our group had developed atom-economical reactions between EBX reagents and diazo compounds.^119,120^ In 2019, we tested the EBZ reagents in this transformation.^47^ A highly enantioselective copper-catalyzed oxyalkynylation of diazo compounds was developed with BOX ligand 23 leading to imidates in moderate to excellent yields *via* an ambiphilic copper-carbene intermediate I (Scheme 42).

**Scheme 43:**
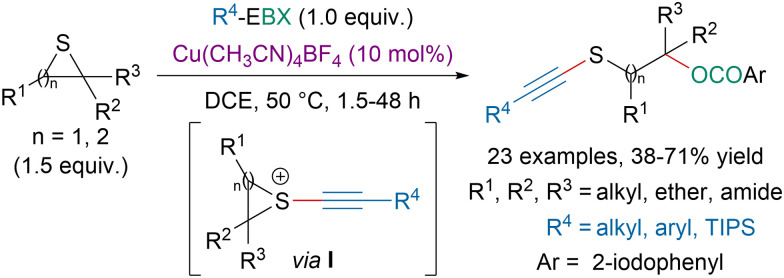
Copper-catalyzed oxyalkynylation of thiiranes and thiethanes.

In 2020, our group disclosed a copper-catalyzed 1,3-oxyalkynylation of thiiranes and 1,4-oxyalkynylation of thiethanes with EBX reagents (Scheme 43).^153^ Literature precedence and mechanistic studies suggested that the reaction proceeds first *via* the activation of EBX reagents by a copper catalyst followed by addition of a sulfide leading to an episulfonium intermediate I. Ring opening by the nucleophilic attack of an aryl carboxylate species would generate the targeted compound.

**Scheme 44:**
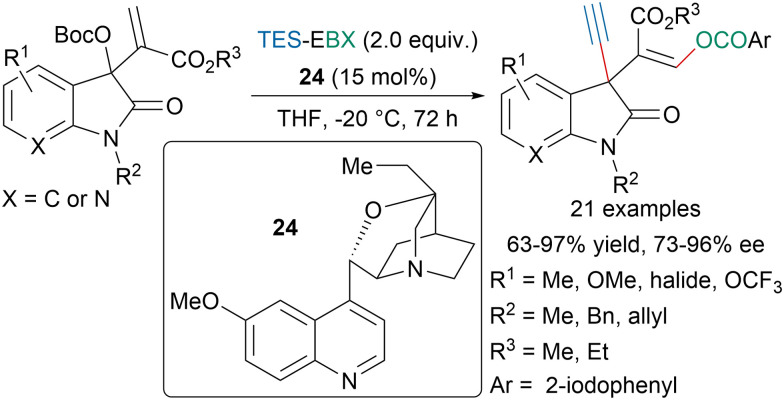
Enantioselective 1,3-oxyalkynylation of MBH isatin carbonates.

Chen, Liang and coworkers developed an enantioselective 1,3-oxyalknylation of Morita–Baylis–Hillman isatin carbonates using a chiral tertiary amine organocatalyst 24 (Scheme 44).^154^ The authors proposed that under the slightly basic conditions TES-EBX would be converted into H-EBX prior to the transfer of the alkyne. With the same strategy they could also develop an enantioselective 1,3-aminosulfenylation from *N*-(arylthio)succinimides.

**Scheme 45:**
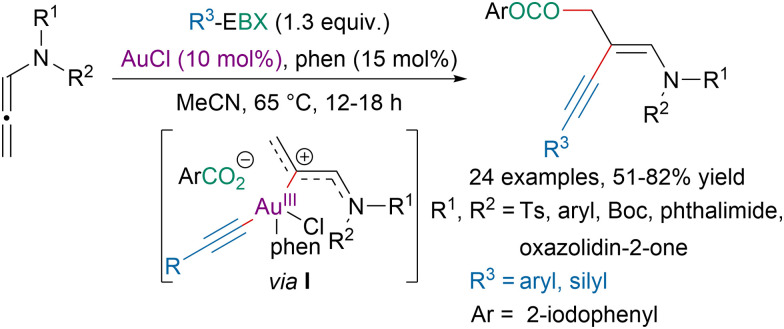
Gold-catalyzed 1,2-oxyalkynylation of *N*-allenamides.

In 2018, the Patil group reported a gold-catalyzed 1,2-oxyalkynylation of *N*-allenamides leading to 1,3-enynes (Scheme 45).^155^ The authors suggested that the reaction would proceed *via* intermediate I obtained after oxidative addition of a gold(i) species to the EBX reagent followed by coordination of the *N*-allenamide. Reductive elimination followed by nucleophilic addition of the aryl carboxylate from the EBX reagent would provide the targeted product. More recently, the same strategy was applied by the Hashmi group for the 1,2-oxyalkynylation of propargylamines leading to highly functionalized alkenes.^156^

**Scheme 46:**
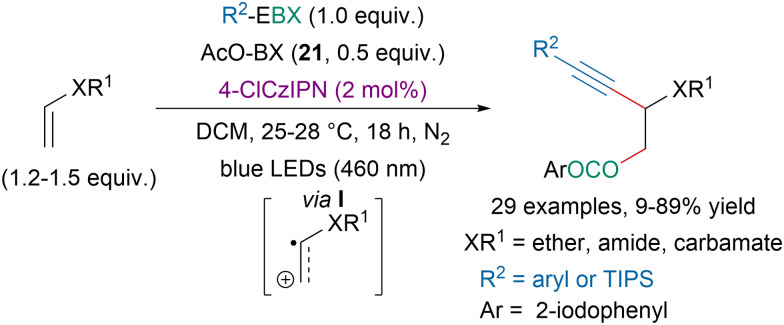
Photoredox-catalyzed oxyalkynylation of enols and enamides.

In 2020, our group reported a photoredox-catalyzed oxyalkynylation of enols and enamides (Scheme 46).^148^ It should be noted that better yields were obtained with an organic dye than *via* direct photoexcitation of EBX reagents (*vide supra*). The reaction is believed to involve the generation of radical cation intermediate I and 21 is suspected to initiate the reaction *via* the formation of iodanyl radicals.

## 7. Conclusions

The significance of alkynes in organic chemistry and other applied fields has been unquestionably established in the last decades. While the introduction of alkynes as nucleophiles into molecules has been the focus of intensive research, the development of electrophilic sources of alkynes has continued to attract more and more attention. Owing to their high reactivity and environmental friendliness, hypervalent iodine reagents have emerged as excellent alkyne electrophilic synthons. The limited stability of alkynyl iodonium salts has initially hampered the broad utilization of these reagents in alkynylation reactions. In contrast, since 2009, the more stable cyclic ethynylbenziodoxolone (EBX) reagents have found widespread applications. In this feature article, we presented the progress since 2018 in the development of new hypervalent iodine reagents, in base- or transition-metal-mediated as well as radical alkynylation transformations and in atom-economical reactions involving hypervalent iodine reagents.

The application of EBX reagents for the functionalization of biomolecules is still in its infancy but could hold great promises for the future. The direct photoexcitation of aryl-EBX reagents allowed to discover and develop new radical alkynylation reactions. The simplicity of this method would make it well suitable for high-throughput experimentations to facilitate the discovery of new exciting transformations. Examples of enantioselective alkynylation reactions with hypervalent iodine reagents are still scarce in the literature and more effort to address this limitation would be needed in the future. Likewise, although EBX reagents have been widely studied in photoredox-catalyzed transformations, their use in electrochemistry is rare and would be worth more thorough investigations. Finally, one intrinsic limitation of these reagents, when used in alkynyl transfer reactions, is the stoichiometric generation of an aryl iodide side product. The development of alkynylation reactions catalytic in an organic iodine would be of high interest.

## Author contributions

E. L. D. wrote and corrected the manuscript. J. W. proofread and edited the manuscript.

## Conflicts of interest

There are no conflicts to declare.

## Supplementary Material
